# Supplementary material for: Enhancing property and activity prediction and interpretation using multiple molecular graph representations with MMGX
Source: Commun Chem. 2024 Apr 5;7:74. doi: 10.1038/s42004-024-01155-w (PMC10997661; doi:10.1038/s42004-024-01155-w)
Supplement: Supplementary file 2 — Supplementary Information [file 42004_2024_1155_MOESM2_ESM.pdf]

Supplementary Information

Enhancing property and activity prediction and interpretation using multiple molecular graph representations with MMGX

Apakorn Kengkanna and Masahito Ohue
School of Computing, Tokyo Institute of Technology
Contact: ohue@c.titech.ac.jp

Supplementary Note 1 - Dataset Description

Table S1 Dataset description

Group	Dataset	Description
**General benchmark datasets from MoleculeNet**	BACE	Binding results in classification label for a set of inhibitors targeting human β-secretase 1 (BACE-1)
	BBBP	Prediction of blood-brain barrier penetration by small molecules in binary label
	FreeSolv	Experimental hydration-free energy for common small molecules in water
	ESOL	Water solubility data (log solubility in mols per litre) for general small molecules
	Lipo	Experimental octanol/water distribution coefficient data for common small molecules (logD at pH 7.4)
**Pharmaceutical endpoint tasks with reported key structural patterns**	AmesMutag	Classification results from Ames test (bacterial reverse mutation assay) to detect mutagenicity in vitro
	hERG20	Experimental assays of hERG (human ether-à-go-go-related gene) blockage bioactivities with unified IC_{50} value (μM)
	CYP2C8	Classification results of inhibitor and noninhibitor targeting CYP2C8 (member of the CYP2C subfamily of cytochrome P450 enzymes)
	CYP3A4	Classification results of inhibitor and noninhibitor targeting CYP3A4 (member of cytochrome P450 enzymes)
	Hepatotoxicity	Binary label of compound toxicity related to liver damage, drug-induced liver injury (DILI)
	ROCKII	Classification ROCK II inhibitors (Rho-associated protein kinases) from experimental IC_{50} values
	HumanPPB	Binding affinity of a drug with plasma proteins or plasma protein binding (PPB) property values
	AqSolDB	Aqueous solubility of compounds (LogS, aqueous solubility in mol/L)
	HIV1	Bioactivity values of HIV-1 Protease inhibitors (human immunodeficiency virus)
	JAK1	Experimental IC_{50} values of JAK1 (Janus kinase)
**Synthetic binding logics with known ground truths**	Logic6	Small molecules containing fluoride and carbonyl substructure (logic: "[FX1] and [CX3]=O")
	Logic7	Small molecules containing unbranched alkane and carbonyl substructure (logic: "[R0;D2,D1][R0;D2][R0;D2,D1] and [CX3]=O")
	Logic9	Small molecules containing logic primary amine and ether and phenyl substructures (logic: "[NX3;H2] and [OD2](C)C and [cX3]1[cX3H][cX3H][cX3H][cX3H][cX3H]1")
	Logic14	Small molecules containing ether or no alcohol, and carbonyl, but no alkyne substructures (logic: "([OD2](C)C or no [OX2H]) and [CX3]=O and (no [CX2]#[CX2])")
	3MR	Small molecules containing 3-member ring substructure(s) (logic: *1**1)

Supplementary Note 2 - Summary of Molecular Graph Representations

This table displays the summary of the characteristics and node and edge feature description of each molecular graph representation.

Table S2 Node and edge features of molecular graph representations

Graph	#Node Features	#Edge Features	Node Features	Edge Features
Atom (A)	79	10	Atom properties	Bond Properties
Pharmacophore (P)	6	3	Predefined node types from Extended Reduced Graphs (ErG)	Types of connected nodes
JunctionTree (J)	83	6	Edges, rings, and intersection atoms with number of each atom	Types of connected nodes
FunctionalGroup (F)	115	20	Predefined types of edges, rings, and functional groups	Types of connected nodes and number of intersections

Supplementary Note 3 - Difference between Molecular Graph Representations

This table shows the node and edge feature sets for each molecular graph representation to exhibit the difference between graphs.

Table S3 Summary of difference between molecular graph representations

Graph	Node Features					Edge Features
	Atom	Bond	Ring	Functional group	Pharma-cophore	Edge
Atom (A)	Any					Chemical Bond
Pharmacophore (P)			Aromatic only		6 Types	Connection type
JunctionTree (J)	Junction Atom	Any (composition)	Any (composition)			Connection type
FunctionalGroup (F)		Predefined bond types	Predefined ring types	Predefined functional groups		Connection type

Supplementary Note 4 - Molecular Graph Reduction Analysis

This graph shows the average number of nodes from all molecules in different molecular graphs from general benchmark datasets.

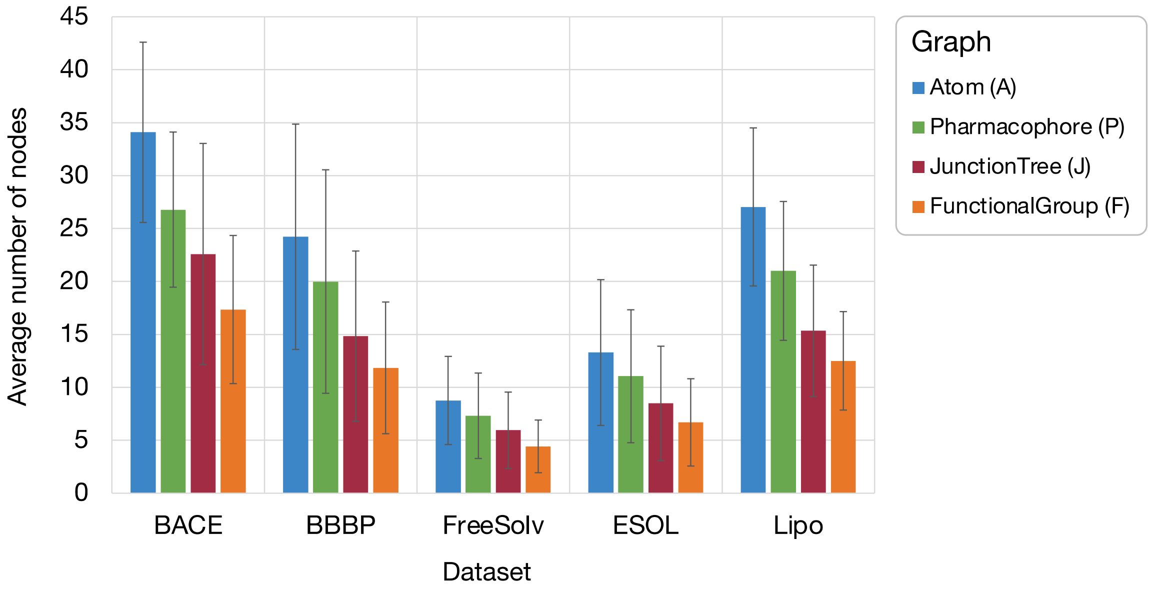

Figure S1 Analysis of average number of nodes between each molecular graph representation. This graph visualizes the average number of nodes in each graph after reduction from all molecules in general benchmark datasets. Error bars represent standard deviation.

Supplementary Note 5 - Model Performance of 2-graph Combination Models

Model performance ranking of 2-graph models for physical chemistry, biophysics, physiology category datasets with MMGX. The model performances are recorded as AUROC for classification tasks (↑) and RMSE for regression tasks (↓).

Table S4 Model performance of physical chemistry category datasets

Model	FreeSolv↓	ESOL↓	Lipophilicity↓	AqSolDB↓	AvgRank	AvgZScore
**A**	1.5053(0.0731)	0.7276(0.0488)	0.5839(0.0139)	1.0115(0.0261)	3.00	-0.6793
**A+F**	**1.2677(0.0826)**	0.7460(0.0570)	0.5926(0.0128)	**0.9816(0.0185)**	2.50	0.1644
**A+P**	1.3744(0.0792)	**0.6761(0.0188)**	0.5915(0.0118)	0.9882(0.0189)	**2.00**	**0.3952**
**A+J**	1.3772(0.0097)	0.7362(0.0307)	**0.5765(0.0189)**	1.0016(0.0197)	2.50	0.1197

The underlined numbers are the best performance of each dataset. The numbers in parenthesis are the standard deviations.

Table S5 Model performance of biophysics category datasets

Model	BACE↑	hERG20↑	CYP2C8↑	CYP3A4↑	ROCKII↑	HumanPPB↓	HIV1↓	JAK1↓	AvgRank	AvgZScore
**A**	0.7090(0.0245)	0.9227(0.0057)	**0.8553(0.0088)**	0.9074(0.0019)	0.9974 (0.0034)	0.1347(0.0083)	1.2817(0.0425)	0.6162(0.0073)	3.13	-0.4856
**A+F**	0.7320(0.0279)	**0.9275(0.0043)**	0.8505(0.0146)	0.9060(0.0029)	0.9990 (0.0012)	0.1389(0.0130)	**0.9914(0.0336)**	**0.5728(0.0095)**	2.25	**0.4613**
**A+P**	0.7412(0.0175)	0.9233(0.0067)	0.8506(0.0202)	**0.9081(0.0023)**	0.9982 (0.0034)	**0.1312(0.0140)**	1.2789(0.0972)	0.5903(0.0073)	**2.00**	0.1481
**A+J**	**0.7483(0.0323)**	0.9231(0.0048)	0.8443(0.0101)	0.9069(0.0027)	**0.9998 (0.0002)**	0.1627(0.0094)	1.1041(0.0499)	0.5907(0.0045)	2.63	-0.1238

The underlined numbers are the best performance of each dataset. The numbers in parenthesis are the standard deviations.

Table S6 Model performance of physiology category datasets

Model	BBBP↑	AmesMutag↑	Hepatotoxicity↑	AvgRank	AvgZScore
**A**	0.8828(0.0187)	0.8604(0.0056)	0.7184(0.0087)	3.67	-0.8079
**A+F**	0.8859(0.0032)	**0.8680(0.0040)**	**0.7724(0.0131)**	**1.33**	**1.0827**
**A+P**	**0.8922(0.0056)**	0.8649(0.0033)	0.7470(0.0118)	1.67	0.6556
**A+J**	0.8747(0.0077)	0.8611(0.0019)	0.7322(0.0187)	3.33	-0.9304

The underlined numbers are the best performance of each dataset. The numbers in parenthesis are the standard deviations.

Supplementary Note 6 - Multiple-graph Combination Models Experiments

To test the effectiveness of multiple molecular graph representations model, the reduced graph only models (F, J, P), the combination of 3-graph models (A+F+J, A+F+P, A+J+P), and the combination of 4-graph models (A+F+P+J) experiments are conducted and tested on general benchmark datasets with MMGX. The model performances are recorded as AUROC for classification tasks (↑) and RMSE for regression tasks (↓).

Table S7 Model performance of multiple-graph combination models on general benchmark datasets

Model	FreeSolv↓	ESOL↓	Lipo↓	BACE↑	BBBP↑	AvgRank	AvgZScore
**A**	1.5053 (0.0731)	0.7276 (0.0488)	0.5839 (0.0139)	0.7090 (0.0245)	0.8828 (0.0187)	6.2	-0.1780
**F**	1.7978 (0.1897)	0.7508 (0.0228)	0.6717 (0.0108)	0.7396 (0.0207)	0.8671 (0.0104)	8.6	-0.7077
**J**	1.6826 (0.2216)	0.8257 (0.0590)	0.6486 (0.0082)	0.7250 (0.0369)	0.8322 (0.0137)	9.8	-1.5051
**P**	1.9721 (0.0529)	0.7673 (0.0264)	0.6670 (0.0367)	0.7234 (0.0169)	0.8753 (0.0054)	9.4	-1.0913
**A+F**	**1.2677 (0.0826)**	0.7460 (0.0570)	0.5926 (0.0128)	0.7320 (0.0279)	0.8859 (0.0032)	5.2	0.3452
**A+J**	1.3772 (0.0097)	0.7362 (0.0307)	0.5765 (0.0189)	**0.7483 (0.0323)**	0.8747 (0.0077)	5.0	0.5243
**A+P**	1.3744 (0.0792)	0.6761 (0.0188)	0.5915 (0.0118)	0.7412 (0.0175)	**0.8922 (0.0056)**	3.8	0.8089
**A+F+J**	1.3078 (0.0598)	0.7896 (0.0337)	0.5964 (0.0074)	0.7354 (0.0271)	0.8776 (0.0110)	6.4	0.0464
**A+F+P**	1.3471 (0.1186)	0.7939 (0.0748)	0.5905 (0.0279)	0.7342 (0.0308)	0.8903 (0.0156)	5.8	0.1648
**A+J+P**	1.3323 (0.0726)	**0.6728 (0.0126)**	0.5761 (0.0149)	0.7482 (0.0190)	0.8782 (0.0165)	**2.8**	**0.8939**
**A+F+P+J**	1.3396 (0.0691)	0.7321 (0.0225)	**0.5760 (0.0069)**	0.7457 (0.0298)	0.8877 (0.0090)	3.0	0.6985

The underlined numbers are the best performance of each dataset. The numbers in parenthesis are the standard deviations.

Supplementary Note 7 - Model Applicability Experiments

To support the applicability of the concept of multiple molecular graph representation for graph-based model learning, additional experiments on different GNN architecture are conducted. The model performances are recorded as AUROC for classification tasks (↑) and RMSE for regression tasks (↓). As a result, integrating multiple graphs demonstrates promising performance in all different architecture. Therefore, the concept of multiple graphs could help improve model performance positively.

Table S8 Model performance of proposed model MMGX on general benchmark datasets

Model	Architecture	FreeSolv↓	ESOL↓	Lipo↓	BACE↑	BBBP↑	AvgRank	AvgZScore
**A**	GIN+GAT	1.5053 (0.0731)	0.7276 (0.0488)	0.5839 (0.0139)	0.7090 (0.0245)	0.8828 (0.0187)	3.00	-0.6256
**A+F**	GIN+GAT	**1.2677 (0.0826)**	0.7460 (0.0570)	0.5926 (0.0128)	0.7320 (0.0279)	0.8859 (0.0032)	2.80	-0.0567
**A+P**	GIN+GAT	1.3744 (0.0792)	**0.6761 (0.0188)**	0.5915 (0.0118)	0.7412 (0.0175)	**0.8922 (0.0056)**	**1.80**	**0.5658**
**A+J**	GIN+GAT	1.3772 (0.0097)	0.7362 (0.0307)	**0.5765 (0.0189)**	**0.7483 (0.0323)**	0.8747 (0.0077)	2.40	0.1165

The underlined numbers are the best performance of each dataset. The numbers in parenthesis are the standard deviations.

Table S9 Model performance of simple Graph Convolutional Network (GCN) model on general benchmark datasets

Model	Architecture	FreeSolv↓	ESOL↓	Lipo↓	BACE↑	BBBP↑	AvgRank	AvgZScore
**GCN_A**	GCN	1.7064 (0.0674)	1.0125 (0.1327)	0.7692 (0.0325)	0.6104 (0.0562)	0.8619 (0.0031)	4.00	-1.6463
**GCN_A+F**	GCN	**1.2836 (0.1226)**	0.7409 (0.0107)	**0.6009 (0.0084)**	0.7424 (0.0134)	0.8806 (0.0119)	**1.60**	**0.7542**
**GCN_A+P**	GCN	1.4675 (0.0721)	**0.7202 (0.0456)**	0.6337 (0.0145)	**0.7489 (0.0172)**	**0.8879 (0.0094)**	**1.60**	0.6200
**GCN_A+J**	GCN	1.4683 (0.0930)	0.7766 (0.0392)	0.6315 (0.0129)	0.7337 (0.0666)	0.8782 (0.0086)	2.80	0.2721

The underlined numbers are the best performance of each dataset. The numbers in parenthesis are the standard deviations.

Table S10 Model performance of AttentiveFP model on general benchmark datasets

Model	Architecture	FreeSolv↓	ESOL↓	Lipo↓	BACE↑	BBBP↑	AvgRank	AvgZScore
**AFP_A**	AttentiveFP	1.8585 (0.2998)	0.6941 (0.0653)	0.6489 (0.0331)	0.7279 (0.0141)	0.8917 (0.0056)	3.40	-0.7807
**AFP_A+F**	AttentiveFP	**1.3492 (0.1188)**	0.7021 (0.0465)	0.6208 (0.0311)	**0.7434 (0.0235)**	0.8795 (0.0063)	2.60	-0.1257
**AFP_A+P**	AttentiveFP	1.5552 (0.1177)	0.6947 (0.0374)	**0.6148 (0.0218)**	0.7361 (0.0208)	**0.8934 (0.0075)**	2.20	0.3641
**AFP_A+J**	AttentiveFP	1.4370 (0.0651)	**0.6921 (0.0491)**	0.6182 (0.0177)	0.7362 (0.0140)	0.8924 (0.0065)	**1.80**	**0.5423**

The underlined numbers are the best performance of each dataset. The numbers in parenthesis are the standard deviations.

Supplementary Note 8 - Mode Performance of Different Model Architectures for All Datasets

Additional experiments of the 2-graph combination model are conducted with GCN and AttentiveFP model architectures across all datasets in pharmaceutical endpoint tasks to support the conclusion of the most promising and recommended combination. The model performances are recorded as AUROC for classification tasks (↑) and RMSE for regression tasks (↓).

Table S11 Model performance of simple Graph Convolutional Network (GCN) model on pharmaceutical endpoint tasks datasets

Model	Architecture	AqSolDB↓	hERG20↑	CYP2C8↑	CYP3A4↑	HumanPPB↓
**GCN_A**	GCN	1.1513 (0.0274)	0.8952 (0.0061)	0.8259 (0.0189)	0.8934 (0.0020)	0.1561 (0.0058)
**GCN_A+F**	GCN	**<u>1.0984 (0.0393)</u>**	**<u>0.9201 (0.0055)</u>**	**<u>0.8568 (0.0153)</u>**	0.8988 (0.0013)	0.1435 (0.0087)
**GCN_A+P**	GCN	1.1544 (0.0170)	0.9077 (0.0041)	0.8520 (0.0171)	**<u>0.9039 (0.0018)</u>**	0.1743 (0.0287)
**GCN_A+J**	GCN	1.1197 (0.0058)	0.9112 (0.0097)	0.8369 (0.0209)	0.9021 (0.0027)	**<u>0.1433 (0.0061)</u>**

Model	Architecture	ROCKII↑	HIV1↓	JAK1↓	AmesMutag↑	Hepatotoxicity↑
**GCN_A**	GCN	**<u>0.9994 (0.0001)</u>**	1.3672 (0.0621)	0.7037 (0.0069)	0.8487 (0.0047)	0.7261 (0.0170)
**GCN_A+F**	GCN	0.9986 (0.0009)	**<u>1.1159 (0.0624)</u>**	0.6377 (0.0055)	0.8568 (0.0042)	**<u>0.7565 (0.0065)</u>**
**GCN_A+P**	GCN	0.9980 (0.0032)	1.2027 (0.0699)	0.6459 (0.0061)	**<u>0.8588 (0.0027)</u>**	0.7355 (0.0222)
**GCN_A+J**	GCN	0.9983 (0.0016)	1.2129 (0.0341)	**<u>0.6340 (0.0038)</u>**	0.8529 (0.0035)	0.7424 (0.0203)

The underlined numbers are the best performance of each dataset. The numbers in parenthesis are the standard deviations.

Table S12 Model performance of AttentiveFP model on pharmaceutical endpoint tasks datasets

Model	Architecture	AqSolDB↓	hERG20↑	CYP2C8↑	CYP3A4↑	HumanPPB↓
**AFP_A**	AttentiveFP	1.0402 (0.0159)	**<u>0.9218 (0.0025)</u>**	0.8390 (0.0128)	0.9095 (0.0024)	**<u>0.1327 (0.0056)</u>**
**AFP_A+F**	AttentiveFP	**<u>1.0231 (0.0306)</u>**	0.9214 (0.0070)	**<u>0.8488 (0.0327)</u>**	0.9054 (0.0013)	0.1416 (0.0095)
**AFP_A+P**	AttentiveFP	1.0246 (0.0193)	0.9178 (0.0031)	0.8471 (0.0062)	**<u>0.9109 (0.0010)</u>**	0.1389 (0.0060)
**AFP_A+J**	AttentiveFP	1.0288 (0.0119)	0.9141 (0.0024)	0.8475 (0.0077)	0.9056 (0.0027)	0.1422 (0.0104)

Model	Architecture	ROCKII↑	HIV1↓	JAK1↓	AmesMutag↑	Hepatotoxicity↑
**AFP_A**	AttentiveFP	0.9989 (0.0002)	1.4310 (0.0805)	0.6392 (0.0071)	0.8585 (0.0046)	0.7288 (0.0107)
**AFP_A+F**	AttentiveFP	**<u>0.9991 (0.0007)</u>**	**<u>1.3596 (0.0202)</u>**	**<u>0.5883 (0.0060)</u>**	**<u>0.8626 (0.0043)</u>**	**<u>0.7524 (0.0105)</u>**
**AFP_A+P**	AttentiveFP	0.9967 (0.0024)	1.4060 (0.0482)	0.6477 (0.0085)	0.8601 (0.0017)	0.7382 (0.0073)
**AFP_A+J**	AttentiveFP	0.9947 (0.0066)	1.3686 (0.0461)	0.6457 (0.0142)	0.8573 (0.0019)	0.7269 (0.0122)

The underlined numbers are the best performance of each dataset. The numbers in parenthesis are the standard deviations.

Supplementary Note 9 - Comparison with Other Models

Five methods are used for comparison including, PharmHGT [7], which utilizes pharmacophoric-constrained heterogeneous graphs employing BRICS fragmentation for generating a pharmacophore view connecting with atom view with different edge types; HimGNN [8], which proposes hierarchical graph representations consisting of atom- and motif-based graphs using a custom motif extraction process; ML-MPNN [9], which creates a multi-level message passing neural network using four full ranges of levels, including nodes, edges, junction-tree subgraphs, and the entire graph; FunQG [10], which constructs a new molecular graph representation using functional groups as building blocks with the theoretical concept of a quotient graph; and RG-MPNN [11], which demonstrates the integration of pharmacophore information hierarchically into atom-level graphs with a message-passing neural network architecture.

The experiments are reproduced from the online available code repository indicated in the respective publications, using the same sets of dataset splittings of the general benchmark dataset from MoleculeNet used in our research. It should be noted that each research has a different preprocessing policy to handle molecule data and model architectures. The model performances are recorded as AUROC for classification tasks (↑) and RMSE for regression tasks (↓). The results are summarized in Table S13, Figure S2, and Figure S3.

Table S13 Model performance comparison with other state-of-the-art methods

Model	FreeSolv↓	ESOL↓	Lipo↓	BACE↑	BBBP↑
**A**	1.5053 (0.0731)	0.7276 (0.0488)	0.5839 (0.0139)	0.7090 (0.0245)	0.8828 (0.0187)
**A+F**	1.2677 (0.0826)	0.7460 (0.0570)	0.5926 (0.0128)	0.7320 (0.0279)	0.8859 (0.0032)
**A+P**	1.3744 (0.0792)	0.6761 (0.0188)	0.5915 (0.0118)	0.7412 (0.0175)	0.8922 (0.0056)
**A+J**	1.3772 (0.0097)	0.7362 (0.0307)	0.5765 (0.0189)	0.7483 (0.0323)	0.8747 (0.0077)
**PharmHGT [7]**	1.0175 (0.0523)	0.6514 (0.0235)	0.6450 (0.0141)	0.7620 (0.0227)	0.8612 (0.0068)
**HimGNN [8]**	1.4433 (0.1032)	1.1034 (0.1128)	0.8179 (0.0815)	0.7028 (0.0160)	0.8263 (0.0281)
**ML-MPNN [9]**	1.1881 (0.1096)	0.5732 (0.0363)	0.5827 (0.0186)	0.7402 (0.0152)	0.8837 (0.0047)
**FunQG [10]**	0.9930 (0.0320)	0.5900 (0.0190)	0.6130 (0.0050)	0.7700 (0.0100)	0.8890 (0.0030)
**RG-MPNN [11]**	1.0738 (0.1422)	0.6996 (0.0387)	0.6336 (0.0098)	0.7274 (0.0116)	0.8278 (0.0466)

The numbers in parenthesis are the standard deviations.

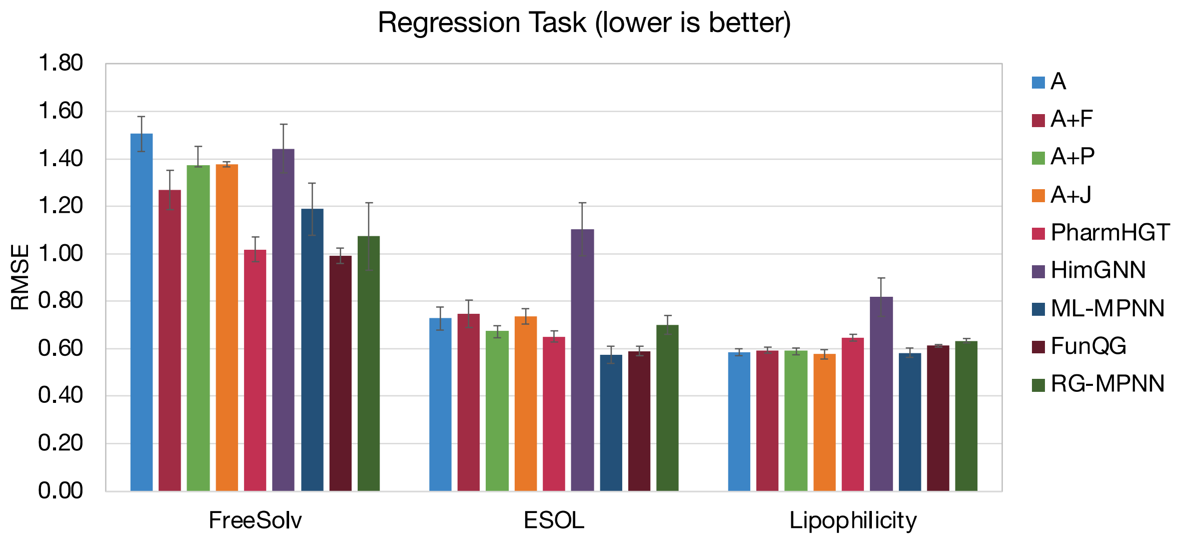

Figure S2 Comparison of model performance between the proposed model and state-of-the-art methods on regression task. Error bars represent standard deviation.

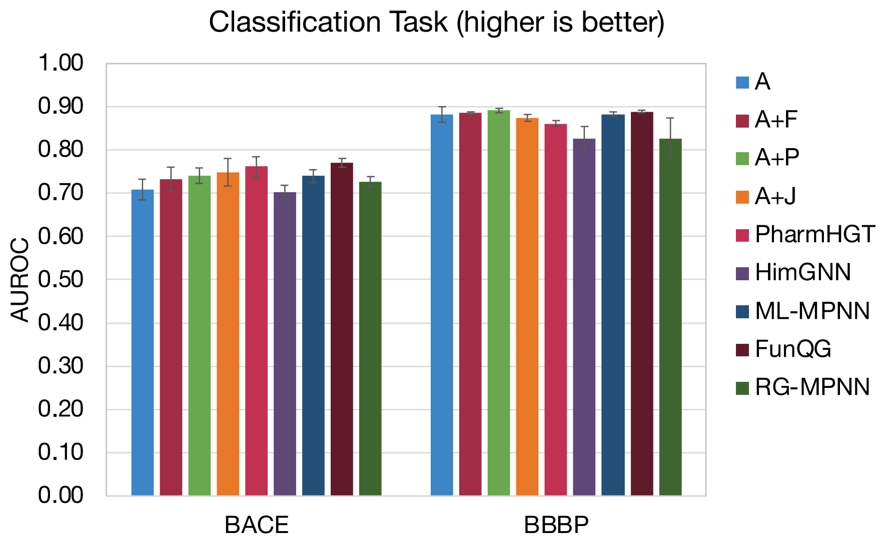

Figure S3 Comparison of model performance between the proposed model and state-of-the-art methods on classification task. Error bars represent standard deviation.

Supplementary Note 10 - Interpretation on Single Prediction View with Ligand Interaction Map

This Figure S4 compares the attention weights with ligand interaction map from BACE dataset. Umibecestat or CNP520, a potent small molecule for BACE-1 inhibitor, is used as an example.

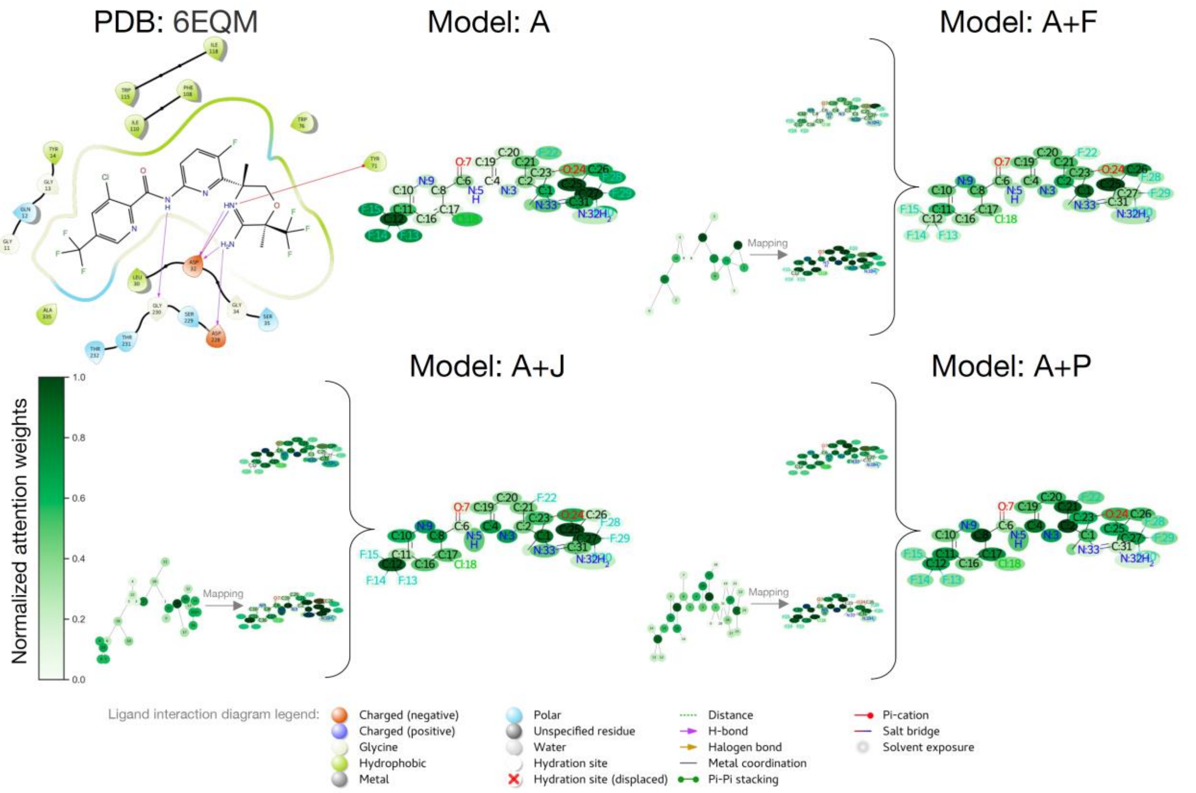

Figure S4 Interpretation on single prediction view of Umibecestat (CNP520) and the interaction map with BACE-1 complex (PDB:6EQM). All visualizations of model attention follow the interpretation extraction procedures. For the 2-graph scheme, the mapping and combining processes are applied to visualize on original atom-level graph. The ligand interaction diagram is calculated and generated by Maestro version 12.4.072, Schrödinger software suite with the legend below.

Supplementary Note 11 - Interpretation on Single Prediction View using Synthetic Binding Logics with Known Ground Truths

This Figure S5 shows the interpretation performance using synthetic binding logics with known ground truths from 3MR (3-member ring) dataset.

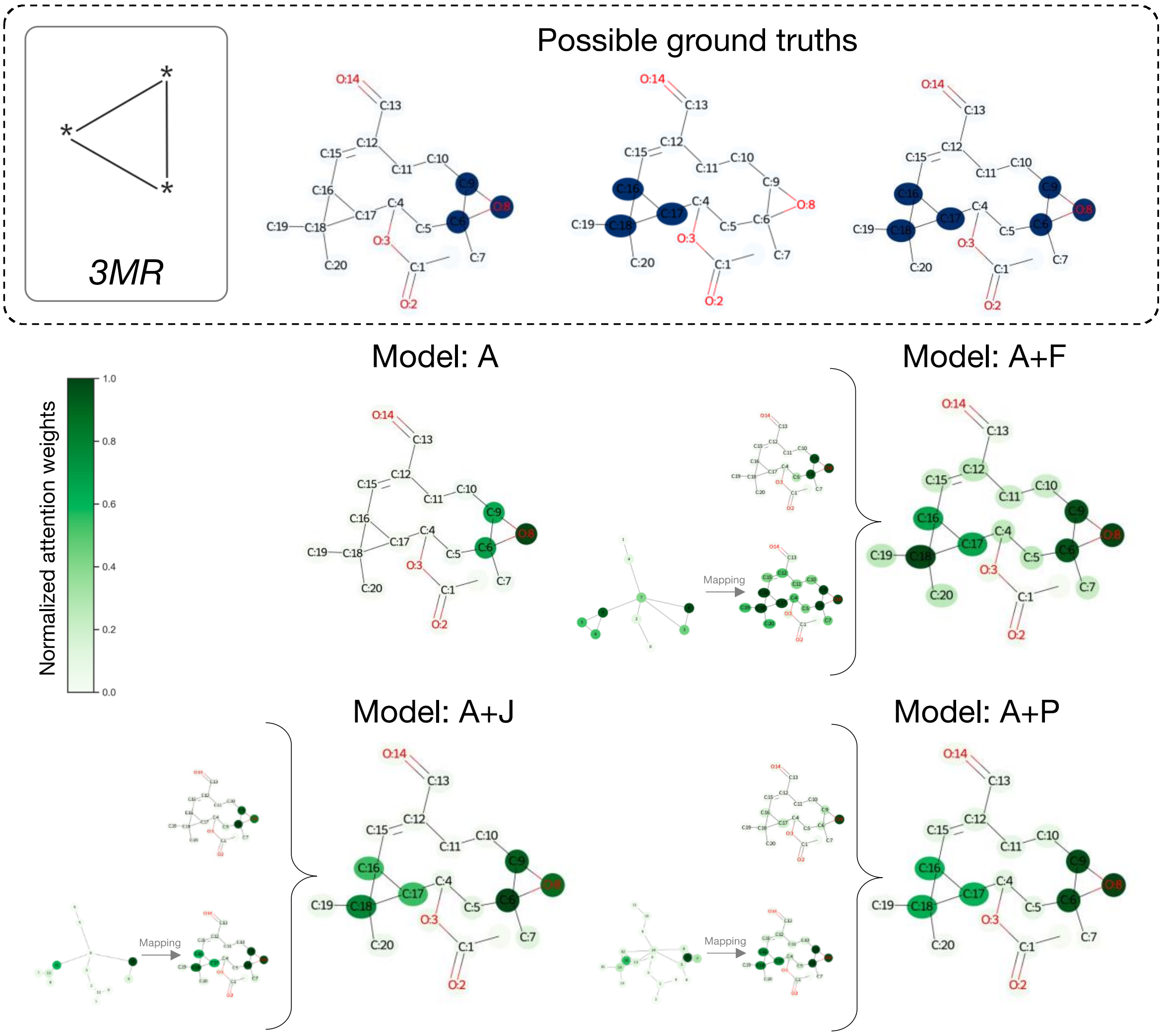

Figure S5 Interpretation on single prediction view of sample molecule from 3MR dataset with all possible ground truths. All visualizations of model attention follow the interpretation extraction procedures. For the 2-graph scheme, the mapping and combining processes are applied to visualize on original atom-level graph.

This Figure S6 shows the interpretation performance using synthetic binding logics with known ground truths from Logic14 dataset.

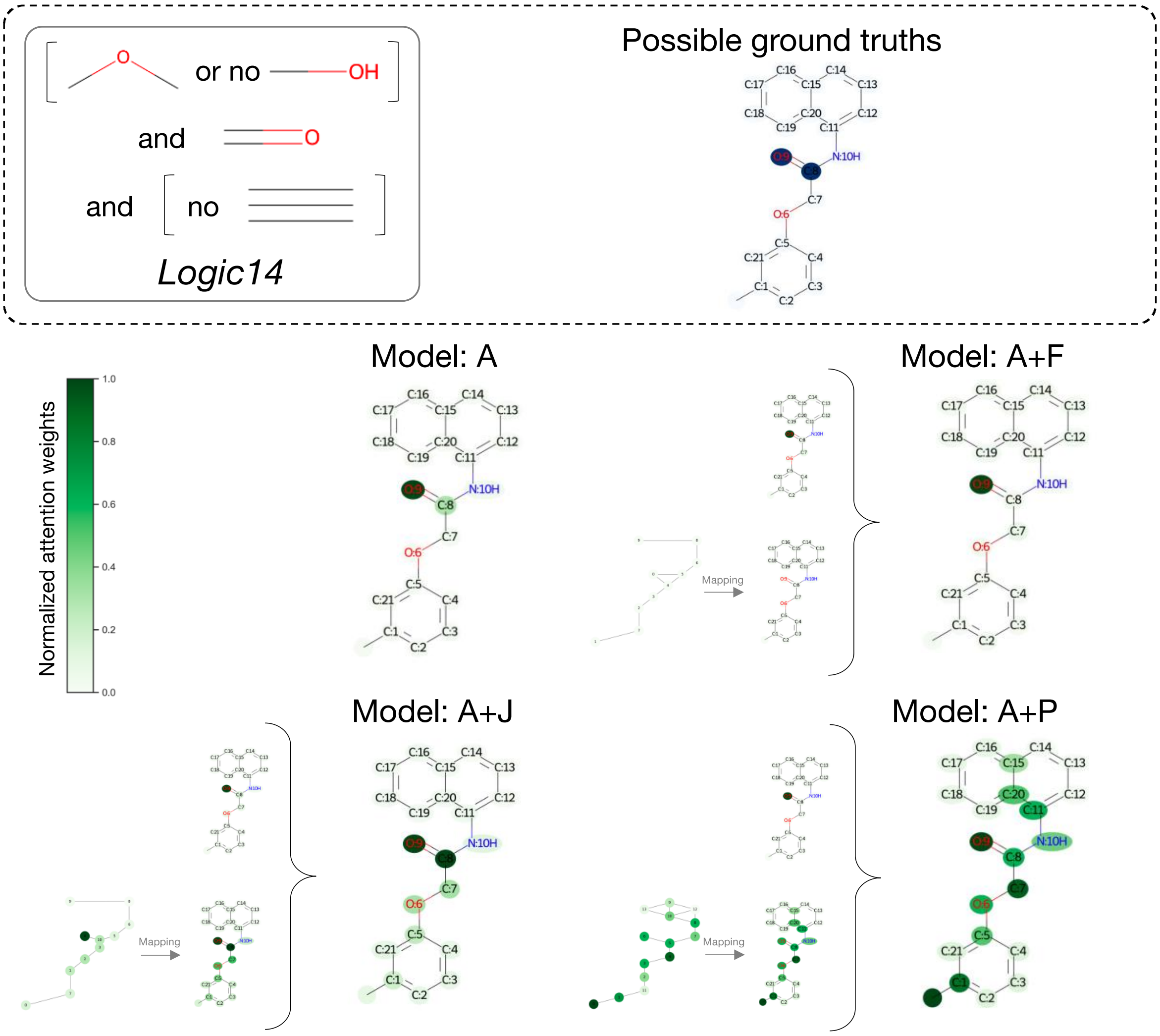

Figure S6 Interpretation on single prediction view of sample molecule from Logic14 dataset with all possible ground truths. All visualizations of model attention follow the interpretation extraction procedures. For the 2-graph scheme, the mapping and combining processes are applied to visualize on original atom-level graph.

Supplementary Note 12 - Analysis of Attention Correlation

Results of attention correlation using attention weights of two graphs from molecule embedding step. Spearman rank-order correlation, a nonparametric measure of the monotonicity of the relationship between two sets of observations, is used for analysis. Most of the models provide weak or even no correlation between two sets of attention from two graphs. These results indicate that models typically focus on different part of molecules. This situation can be considered beneficial, as several important regions in compound can be captured and emphasized via combining process when integrating multiple graphs.

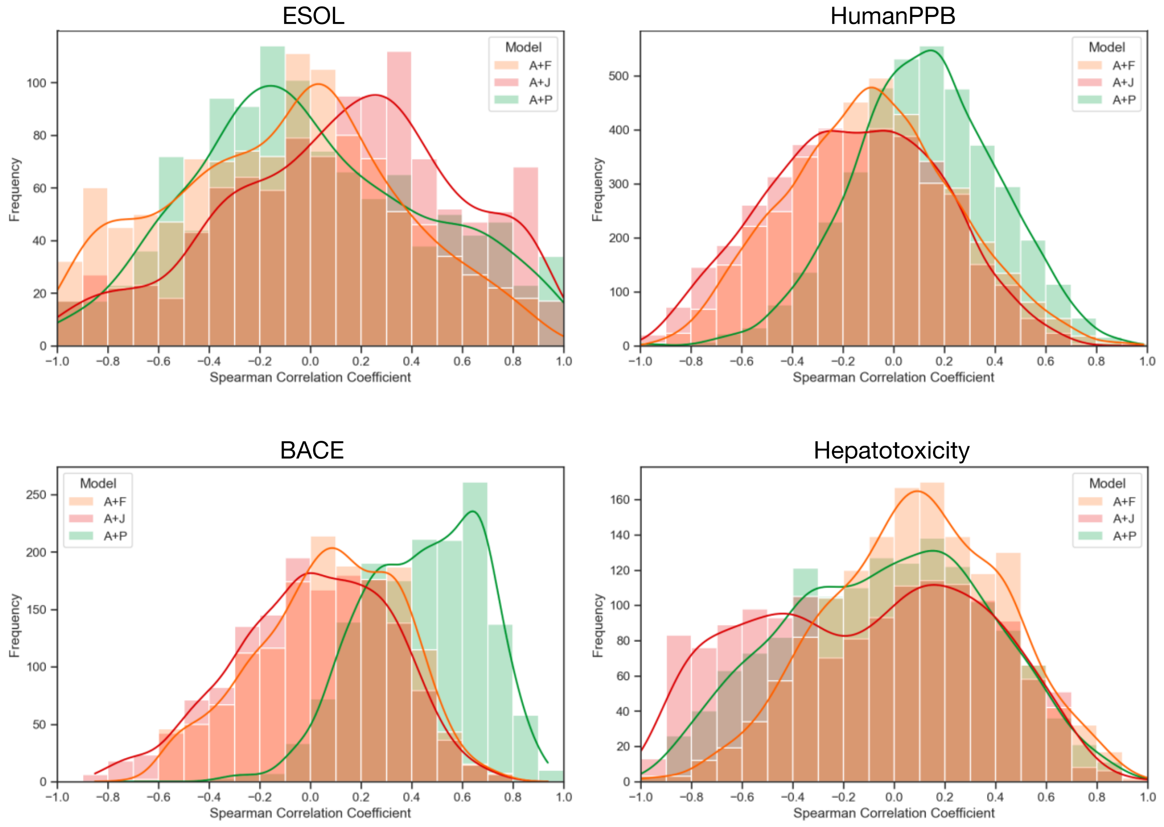

Figure S7 Histogram of attention correlation from all molecules in dataset extracted from attention weights at molecule embedding module between Atom graph and reduced graph based on each schema. The correlation values are calculated using Spearman rank-order correlation coefficient. The lines are computed using kernel density estimate to smooth the distribution.

Supplementary Note 13 - Interpretation on Potential Substructures View with Reported Key Structural Patterns

This Figure S8 shows potential substructures extracted from different model compared with reported key structural patterns from the literature.

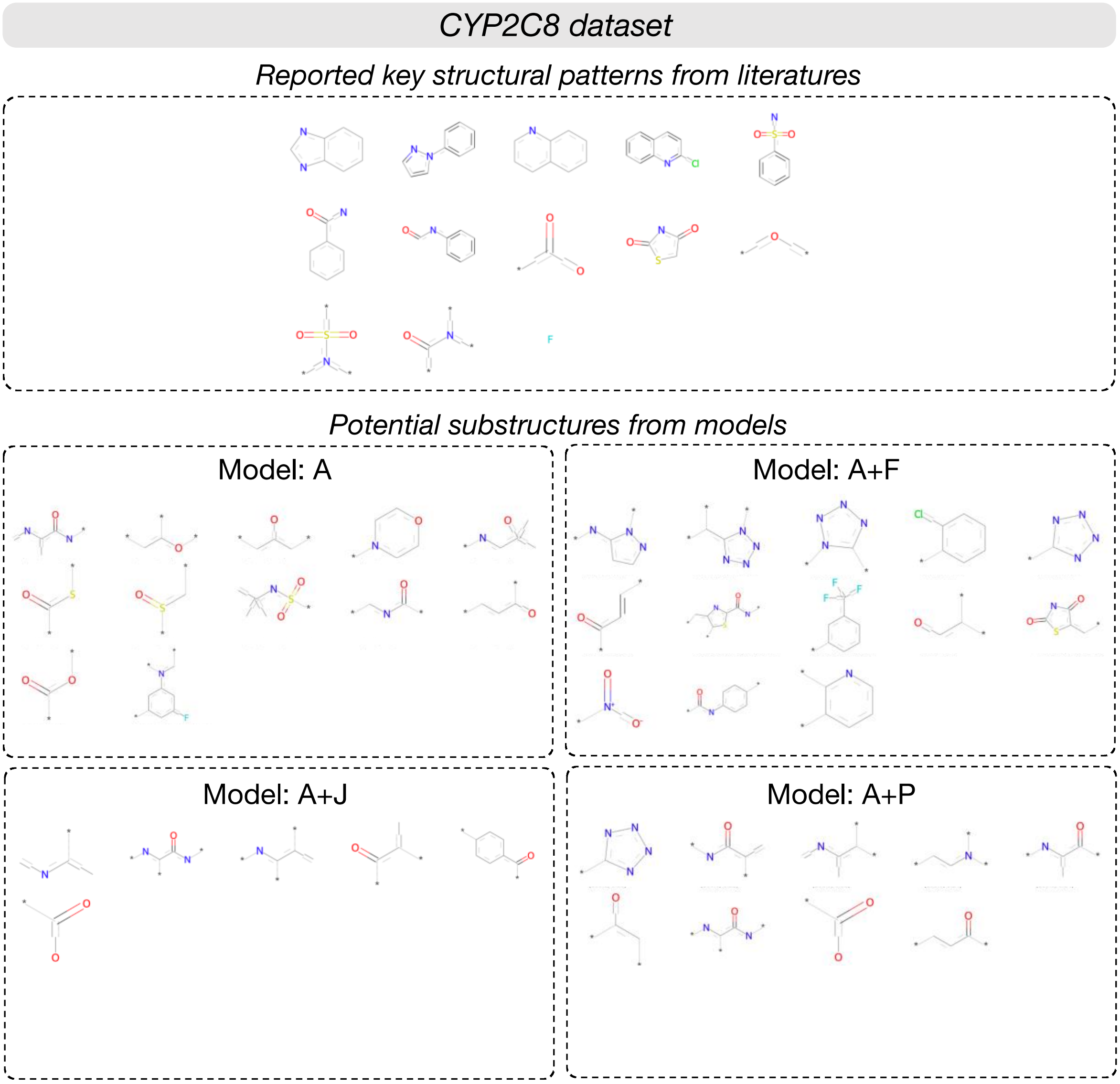

Figure S8 Interpretation on potential substructures view of CYP2C8 dataset. Reported key structural patterns from the literature of CYP2C8 dataset and potential substructures extracted from the models.

Supplementary Note 14 - Molecular Graph Representation Features

The molecule graphs are encoded by node and edge features. Most features are encoded in a one-hot fashion, some are numeric value due to additive nature [1]. The full list of features is displayed in below tables.

S14.1 Atom Graph (A)

Node features (79 features)

Index	Description
0-43	Atom's symbol (from DeepChem [2])
44-54	Atom's degree (0-10)
55-61	Atom's implicit valance
62	Atom's formal charge
63	Atom's number of radical electron
64-68	Atom's hybridization (sp, sp2, sp3, sp3d, sp3d2)
69	Atom's aromatic property
70-74	Atom's total number of Hs (explicit and implicit) (0-4)
75-76	Atom's chiral code (_CIPCode) (R, S)
77	Atom's possible stereocenters (_ChiralityPossible)
78	Atom's in ring property

Edge features (10 features)

Index	Description
0-3	Bond's type (single, double, triple, aromatic)
4	Bond's conjugated property
5	Bond's in ring property
6-9	Bond's stereo type<br>(STEREONONE, STEREOANY, STEREOZ, STEREOE)

S14.2 Pharmacophore Graph (P)

Node features (6 features) [5]

Index	Description
0	Is H-donor
1	Is H-acceptor
2	Is positive
3	Is negative
4	Is hydrophobic
5	Is aromatic

Edge features (3 features)

Index	Description
0-2	Edge connection type (none-none, none-pharmacophore,<br>pharmacophore-pharmacophore)

S14.3 JunctionTree Graph (J)

Node features (83 features) [6]

Index	Description
0-10	Number of atoms (0-10)
11-21	Number of edges (degree) (0-10)
22-32	Number of Hs (0-10)
33	In ring
34	Aromatic
35-78	Number of each atom symbol (from DeepChem [2])
79-82	Number of each bond type (single, double, triple, aromatic)

Edge features (6 features)

Index	Description
0-5	Edge connection type (atom-atom, bond-bond, ring-ring, atom-bond, atom-ring, bond-ring)

S14.4 FunctionalGroup Graph (F)

Node features (115 features)

Index	Description
0-38	Predefined functional group (from RDKit [3])
39-60	Predefined ring type (inspired OpenBabel [4]) with considering aromatic
61-82	Predefined ring type (inspired OpenBabel [4]) without considering aromatic
83-90	Other undefined ring type (3-9, >9)
91-114	Predefined bond type (C-C, C=C, C#C, C-O, C=O, C-N, C=N, C#N, C-S, C=S, C#S, O-O, O-N, O=N, O-S, N-N, N=N, N-S, N#S, N=S, S-S, S=S, S#S, Others)

Edge features (20 features)

Index	Description
0-9	Edge connection type (atom, bond, ring, functional group)
10-19	Number of intersection atom

Supplementary Note 15 - Model Architecture and Interpretation

This Figure S9 shows the illustration of model architecture and interpretation.

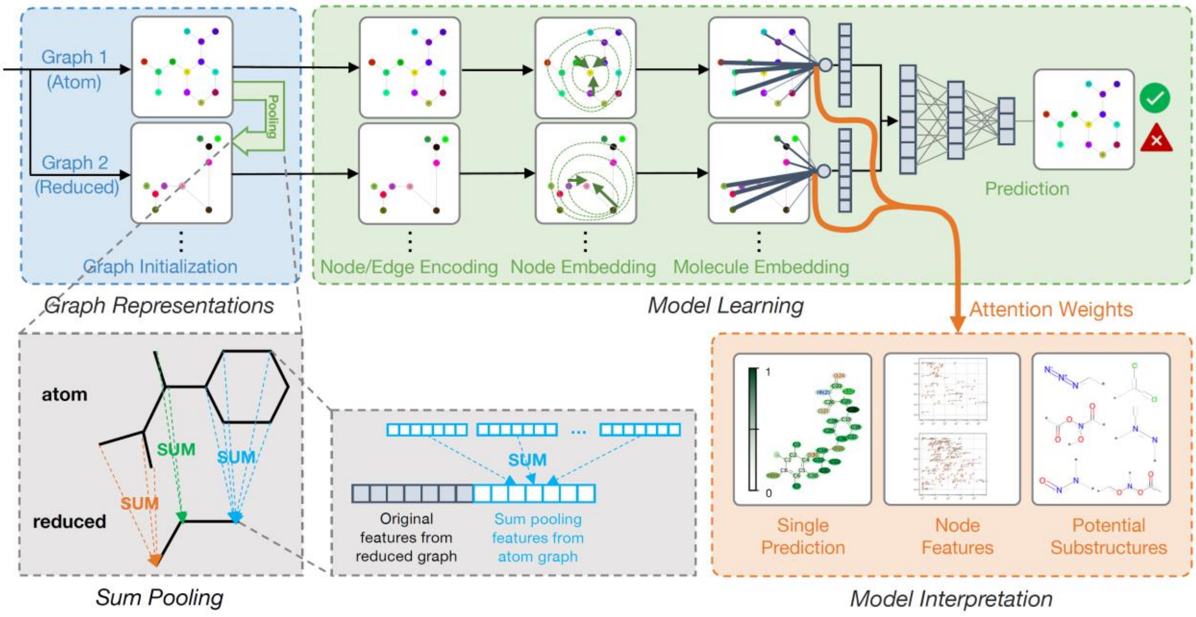

Figure S9 Illustration of model architecture and interpretation. Before proceeding to model learning, the node features of the reduced graph(s) are enriched with information from the corresponding node features of the atom-level graph using the sum pooling method. The model learning process includes node/edge encoding, node embedding using GNN, molecule embedding with attention mechanism, and prediction module. The attention weights from the molecule embedding module are utilized for model interpretation in three different views.

Supplementary Note 16 - Hyperparameter

The models have been performed hyperparameter tuning with the list of parameters displayed in below table.

Table S14 List of hyperparameters

No	Hyperparameter	Range
1	Batch size	16, 32, 64, 128, 256
2	Hidden Layer (All learning blocks)	32, 64, 128, 256
3	Number of node learning layers (for atom graph)	2, 3, 4
4	Number of molecule learning layers (for atom graph)	2, 3, 4
5	Number of node learning layers (for reduced graph)	2, 3, 4
6	Number of molecule learning layers (for reduced graph)	2, 3, 4

Supplementary Note 17 - Interpretation Extraction Procedures

This Figure S10 shows procedures of interpretation extraction from the attention weights.

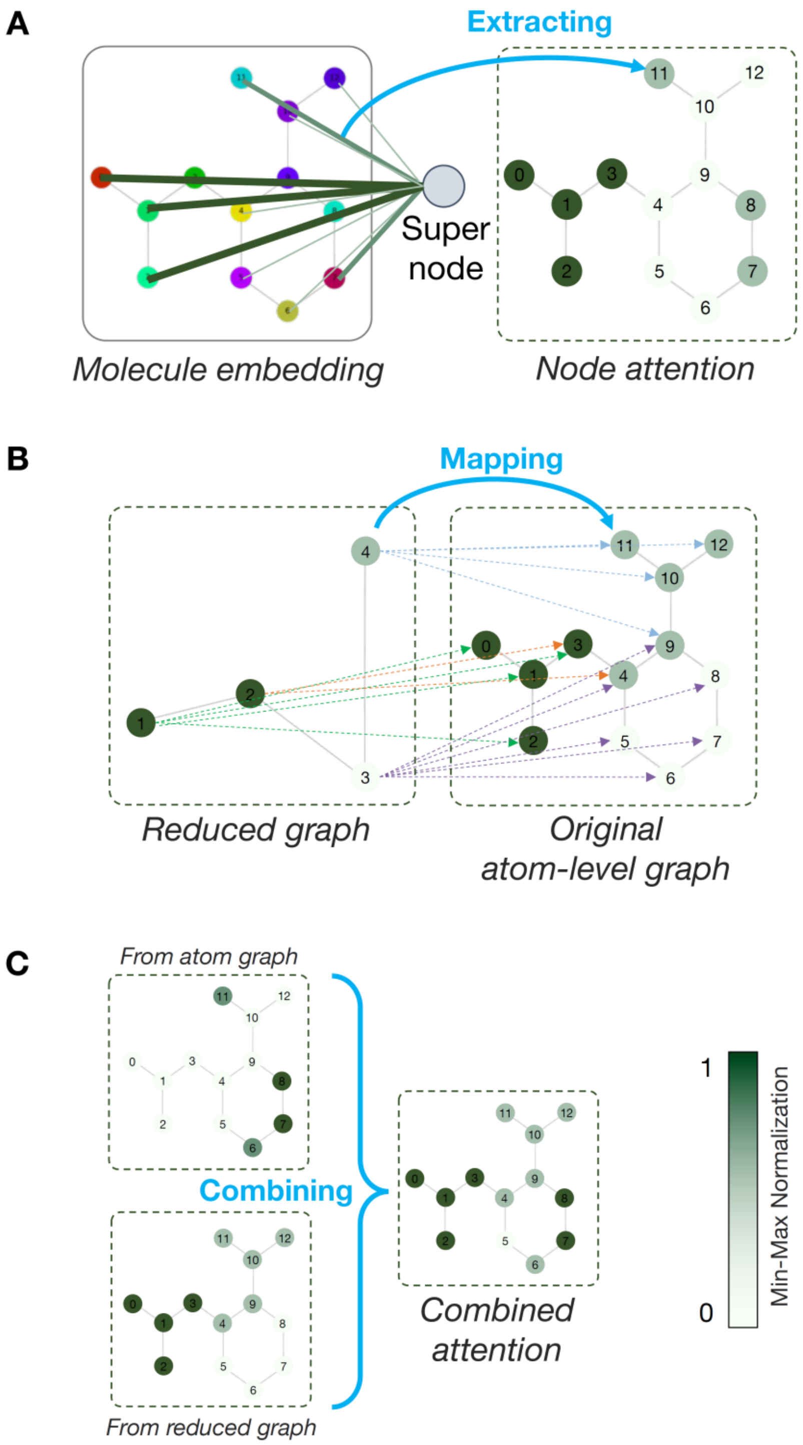

Figure S10 Attention extraction procedures. A) Extracting attentions from molecule embedding from virtual supernode. B) Mapping and distributing attentions from reduced graph into original atom-level graph. C) Combining attention from multiple graphs resulting in final combined attention on atom-level graph.

Supplementary References

[1] Z. Xiong, D. Wang, X. Liu, F. Zhong, X. Wan, X. Li, Z. Li, X. Luo, K. Chen, H. Jiang, and M. Zheng, “Pushing the boundaries of molecular representation for drug discovery with graph attention mechanism,” *J. Med. Chem.*, vol. 63, no. 16, pp. 8749–8760 (2020)

[2] deepchem, “deepchem/graph_features.py at master · deepchem/deepchem,” GitHub, 2015. https://github.com/deepchem/deepchem/blob/master/deepchem/feat/graph_features.py (accessed Jun. 06, 2023).

[3] rdkit, “rdkit/FunctionalGroups.txt at master · rdkit/rdkit,” GitHub, 2013. https://github.com/rdkit/rdkit/blob/master/Data/FunctionalGroups.txt (accessed Jun. 06, 2023).

[4] openbabel, “openbabel/ringtyp.txt at master · openbabel/openbabel,” GitHub, 2015. https://github.com/openbabel/openbabel/blob/master/data/ringtyp.txt (accessed Jun. 06, 2023).

[5] rdkit, “rdkit/ReducedGraphs.cpp at master · rdkit/rdkit,” GitHub, 2013. https://github.com/rdkit/rdkit/blob/master/Code/GraphMol/ReducedGraphs/ReducedGraphs.cpp (accessed Jun. 06, 2023).

[6] W. Jin, R. Barzilay, and T. Jaakkola, “Junction Tree Variational Autoencoder for Molecular Graph Generation,” In *Proc. ICML 2018*, pp.3632–3648 (2018)

[7] Jiang, Y., Jin, S., Jin, X., Xiao, X., Wu, W., Liu, X., Zhang, Q., Zeng, X., Yang, G., Niu, Z.: Pharmacophoric-constrained heterogeneous graph transformer model for molecular property prediction. *Commun. Chem*. 6, 60 (2023)

[8] Han, S., Fu, H., Wu, Y., Zhao, G., Song, Z., Huang, F., Zhang, F., Liu, S., Zhang W.: HimGNN: a novel hierarchical molecular graph representation learning framework for property prediction. *Brief. Bioinform*. 24(5), bbad305 (2023)

[9] Wang, Z., Liu, M., Luo, Y., Xu, Z., Xie, Y., Wang, L., Cai, L., Qi, Q., Yuan, Z., Yang, T., Ji, S.: Advanced graph and sequence neural networks for molecular property prediction and drug discovery. *Bioinformatics*. 38, 2579-2586 (2022)

[10] Hajiabolhassan, H., Taheri, Z., Hojatnia, A., Yeganeh, Y.T.: FunQG: Molecular Representation Learning via Quotient Graphs. *J. Chem. Inf. Model.* 63(11), 3275-3287 (2023)

[11] Kong, Y., Zhao, X., Liu, R., Yang, Z., Yin, H., Zhao, B., Wang, J., Qin, B., Yan, A.: Integrating Concept of Pharmacophore with Graph Neural Networks for Chemical Property Prediction and Interpretation. *J. Cheminform*. 14, 52 (2022)
